# The effect of the Covid-19 Pandemic on pediatric urology

**DOI:** 10.1590/S1677-5538.IBJU.2020.S112

**Published:** 2020-07-27

**Authors:** Anna Bujons Tur, Juan Carlos Prieto, Andrés Gómez-Fraile, Juan Pablo Corbetta

**Affiliations:** 1 Fundació Puigvert Barcelona Division Urology Pediatric Department of Urology Spain Department of Urology, Division Urology Pediatric, Fundació Puigvert Barcelona, Spain; 2 University of Texas Health Science Center Children's Hospital of San Antonio and Methodist Children's Hospital Division Urology Pediatric San AntonioTX USA Division Urology Pediatric, University of Texas Health Science Center, Children's Hospital of San Antonio and Methodist Children's Hospital, San Antonio, TX, USA; 3 Hospital 12 de Octubre Pediatric Urology Division Department of Pediatric Surgery Madrid Spain Department of Pediatric Surgery, Pediatric Urology Division, Hospital 12 de Octubre, Madrid, Spain; 4 Hospital Prof. Dr. Juan P. Garrahan Department of Pediatric Urology Buenos Aires Argentina Department of Pediatric Urology, Hospital Prof. Dr. Juan P. Garrahan, Buenos Aires, Argentina

**Keywords:** Outpatients, Child, COVID-19 [Supplementary Concept], Pandemics

## Abstract

Medical and surgical priorities have changed dramatically at the time of this pandemic. Scientific societies around the World have provided rapid guidance, underpinned by the best knowledge available, on the adaptation of their guidelines recommendations to the current situation. There are very limited scientific evidence especially in our subspecialty of pediatric urology. We carry out a review of the little scientific evidence based mainly on the few publications available to date and on the recommendations of the main scientific societies regarding which patients should undergo surgery, when surgery should be performed and how patient visits should be organize.

## INTRODUCTION

Since the COVID-19 epidemic was first declared in China in December 2019 (1), the virus has spread rapidly around the World owing to its characteristics: rapid spread, high contagiousness, and mortality from viral pneumonia. Critically, hospitals in many countries have had to transform. In Europe as of April 28, there have been 880,000 cases of COVID-19, and specifically in Spain 213,000 cases have been confirmed by Polymerase Chain Reaction (PCR) (2). We have had to convert the departments of our hospitals in an attempt to ensure that human resources and medical infrastructure were adequate to treat patients affected by COVID-19, and a key element of these efforts has been an increase in staff levels through the involvement of doctors from different specialties in the care of these patients. As our healthcare system has become increasingly saturated, most nurses have been moved to COVID-19 areas and the majority of the OR personnel have been moved to the ICU owing to the rise in the need for ventilated beds; these changes have entailed the added difficulty of obtaining adequate personal protective equipment (PPE). Other specialized hospitals have been declared COVID-19 free in order to allow treatment of all patients considered non-infected. Pregnant COVID-19 patients have been transferred to these centers, and all cases of pediatric disease are being treated exclusively in maternal and pediatric hospitals. The COVID-19 crisis has forced health care providers to establish priorities for the treatment of pathologies and to suspend elective surgeries, all with the aim of increasing the number of personnel, and this, too, has meant an involuntary change in our health care systems (3). The decision on which type of care should be postponed and which should continue will need to be reviewed as the pandemic situation changes.

Medical and surgical societies around the World have provided rapid guidance, underpinned by the best knowledge available, on the adaptation of their guidelines recommendations to the current situation (4). But we must also ask ourselves what strategy to follow for those COVID-19 patients who require surgical interventions, bearing in mind the very limited scientific evidence currently available, especially in our subspecialty of pediatric urology.

Here, we carry out a review of the scant scientific evidence based mainly on the few publications available to date and on the recommendations of the main scientific societies regarding which patients should undergo surgery, when surgery should be performed, how patient visits should be organized, which risks need to be addressed, which surgical techniques are safer in this pandemic, how we should protect ourselves, and what risks a child faces when undergoing an operation affected by COVID-19.

## SPECIAL CONSIDERATIONS IN CHILDREN PEDIATRIC PATIENTS, COVID-19 INFECTION, AND COMORBIDITIES

The rapid spread of coronavirus disease 2019 (COVID-19) caused by severe acute respiratory syndrome coronavirus 2 (SARS-CoV-2) has led to a global pandemic, with infection of individuals of all ages residing in almost every country in the World (5). The pediatric population appears to be affected in much smaller proportions than adults, with only 2% of cases described in patients under age 20. According to data published by the Chinese Center for Disease Control and Prevention, only 1% of cases occur in those aged between 10 and 19 years and l% in children under 10 years old (6).

An epidemiologic report described 731 confirmed COVID-19 cases in the pediatric population, with over 90% of patients characterized as asymptomatic or as having mild or moderate symptoms (7). In more severe cases, symptoms can include gastrointestinal symptoms and patients can progress to respiratory failure, shock, coagulation dysfunction, renal damage, septic shock, and multiorgan failure. A case of Kawasaki disease with concurrent COVID-19 infection has recently been published in the literature (8), and cutaneous manifestations of COVID-19 have also been reported in children (9).

As we know, at the moment there is no specific treatment. Symptomatic treatment is administered in mild and moderate cases, with supportive measures and/or treatment of complications in severe cases. Numerous controlled clinical trials with newly developed molecules and drugs already authorized for other indications have been launched for complicated cases, primarily within hospitals (10).

In Spain, the above-described trend continued in April: as of 3 April there were 111 confirmed cases in children under 2 years of age (0.2%), 39 in children aged between 2 and 4 years (0.1%), and 193 in children aged between 5 and 14 years (0.3%). Data were extracted from 54% of the reported cases (63,002 cases) as of that date (117,710 cases) (11). Based on the currently available data, children with COVID-19 have a better prognosis than adults, with few reported severe cases, and in mild cases recovery occurs within 1-2 weeks of disease onset. Most of the confirmed cases were secondary to exposure to family contacts. However, transmission from children to adults and other children can occur, as documented in a number of pediatric cases in China. On the other hand, it has been reported that the elimination of the virus in respiratory secretions and feces occurs over a longer period in children with mild symptoms than in adults, a fact that represents a great challenge for infection control (12). Transmission of the virus from asymptomatic children and a carrier period of up to 21 days have also been demonstrated. These data may explain a greater number of infections. Therefore, children should participate in the usual preventive actions to contain spread of the infection, and the protection of health professionals during evaluation and examination of children with respiratory infections is crucial (11).

While most cases of COVID-19 in children are not severe, there is a population with higher risk factors for poor disease course (Table-1) (9). One of the questions frequently asked by our urological pediatric patients or parents is whether they are at higher risk of suffering from COVID-19 due to their congenital urological diseases the fact that up to now there is no scientific evidence that congenital uropathy is a risk factor for poor evolution for the development of complications in patients with SARS-COVID-2.

**Table 1:** Groups of children at higher risk for poor disease course (Spanish Association of Pediatrics).

Immunosuppressed child	Primary immunodeficiencies (1) Solid organ transplant and hematopoietic progenitor transplantation Treatment with chemotherapy, immunosuppressants, or biological drugs Poorly controlled HIV (detectable CV, CD4 decrease, or CD4/CD8 inversion ratio)
Heart disease	With hemodynamic repercussions With requirement for medical treatment Pulmonary hypertension On transplant waiting list Recent surgery or catheterization
Chronic respiratory pathology (chronic lung diseases)	Cystic fibrosis Bronchopulmonary dysplasia Severe asthma Under tracheostomy, oxygen therapy, or home mechanical ventilation
Others	Dialysis Sickle cell disease Type 1 diabetes mellitus with poor metabolic control Severe malnutrition, short bowel, epidermolysis bullosa, severe encephalopathies, myopathies, inborn errors of metabolism, etc.

When a patient is admitted to our pediatric urology unit and we suspect COVID-19 due to fever or suggestive symptoms, we carry out the PCR test with a pharyngeal sample to rule it out, as well as blood tests (hemogram, coagulation, venous blood gas, biochemistry with LDH, PCR, and PCT) and chest radiography (ideally portable). The use of chest ultrasound should be considered if it is available and if trained personnel are available to perform it. A single family member or other companion authorized by the parents must remain with the patient at all times, complying with the recommended isolation measures (surgical mask, gown, and frequent hand washing). It is recommended that the companion always be the same. In patients with severe disease, measurement of CPK, troponin, BNP, fibrinogen, D-dimer, and ferritin levels is recommended, as well as the acquisition of other data on hemophagocytic lympho- -histiocytosis. Need for lumbar puncture will be assessed if neurological symptoms arise. Indication for other complementary tests will be evaluated according to the circumstances in each case.

## SPECIAL CONSIDERATIONS IN THE SELECTION OF CARE FOR UROPEDIATRIC PATIENTS

Scientific societies for urology, such as the European Association of Urology (EAU) and the American Urological Association (AUA) (13), have created their own information centers for COVID-19 where they can be consulted.

The American College of Surgeons has established basic principles for clinical practice during this period. They recommend minimization of exposure to the hospital environment, with the following guiding principles:

- The goal is to provide timely surgical care to children with emergent and urgent pediatric surgical issues while optimizing patient care resources (e.g., hospital and intensive care unit beds, personal protective equipment, ventilators) and preserving the health of caregivers.
- There is no substitute for sound surgical judgment.
- Surgery should be performed only if delaying the procedure is likely to prolong hospital stay, increase the likelihood of later hospital admission, or cause harm to the patient.
- Children who have failed attempts at medical management of a surgical condition should be considered for surgery to decrease the future use of resources (e.g., recurrent infections in a branchial cleft cyst following a course of antibiotics).
- Shared multidisciplinary decisions regarding surgical scheduling should be made in the context of available institutional resources that will be variable and rapidly evolving.
- Telemedicine and teleconsultation services should be used for patient and physician interaction when available. For this, the creation of local review committees for decision-making related to COVID-19 surgical triage is very important (14).

## URGENT AND ELECTIVE SURGERIES

When the term “urgent surgery” is applied in the specialty of urology, and specifically in adult patients, one most commonly thinks of surgeries for oncological conditions or obstructive urolithiasis with risk of sepsis, which are much less frequent in children. On the other hand, within pediatric urology, one might think of testicular torsions as requiring urgent surgery, or of Wilms tumors, but these are much less frequent than the indications in adults. Most of our patients have congenital pathologies, and in our day-to-day practice we perform mostly reconstructive surgeries, although we can also treat obstructive lithiasis or pathologies involving risk of loss of kidney function. Prioritizing what is urgent or “elective” in this context may be more difficult than in adults. Due to these problems, European societies such as British Association of Pediatrics Urologist (BAPU) and the EAU/ESPU have published recommendations for pediatric urological procedures (Table-2) (4).

**Table 2:** Recommendations from the EAU/ESPU Paediatric Urology Guidelines Panel applicable during the COVID-19 pandemic.

Priority category	Low priority	Intermediate priority	High priority	Emergency
Definition	Clinical harm very unlikely if postponed 6 months	Clinical harm possible if postponed 3-4 months but unlikely	Clinical harm very likely if postponed >6 weeks.	Life-threatening situation
COVID recommendation	Benign scrotal and penile pathology, incontinence.	Semiurgent cases like initial postoperative ultrasound after upper tract surgery.	Urgent cases in which delay may cause irreversible progression or organ damage: includes ultrasound, VCUG in suspected severely obstructed uropathy where surgery is still considered.	Continue all care in which delay is potentially organ threatening or life threatening.
Postoperative follow -up schedule after surgery				
Priority category	Low priority	Intermediate priority	High priority	Emergency
Definition	Clinical harm very unlikely if postponed 6 months.	Clinical harm possible if postponed 3-4 months but unlikely.	Clinical harm very likely if postponed >6 weeks.	Life-threatening situation.
COVID recommendation	Follow-up by 6 months	Follow-up before end of 3 months	Follow-up within <6 weeks.	Follow-up within <24 hr.
	Orchidopexy, hydrocele, hypospadias, circumcision, inguinal hernia, buried penis, urolithiasis if no obstruction or infection.	Any kind of antireflux surgery, pyeloplasty, incontinence surgery if bladder emptying is working	Pyeloplasty with possible loss of function. Recurrent UTI after antireflux surgery. Incontinence surgery with bladder-emptying problems.	Macroscopic hematuria after trauma. Inguinal hernia repair with onset of scrotal pain. Suspected bowel obstruction or intestinal perforation in conjunction with bladder augmentation. Urolithiasis with signs of sepsis and/or obstruction. PUV with urinary retention. Local wound infection or abscess formation after any kind of surgery. Febrile UTI/uroseptical signs after any kind of surgery.
Surgical procedures for pediatric urology cases				
Priority category	Low priority	Intermediate priority	High priority	Emergency
Definition	Clinical harm very unlikely if postponed 6 months	Clinical harm possible if postponed 3-4 months but unlikely	Clinical harm very likely if postponed >6 weeks	Life-threatening situation
COVID recommendation	Defer by 6 months	Treat before end of 3 months Perform surgery that is semiurgent.	Treat within <6 weeks Perform surgery for urgent cases in which delay will cause irreversible progression of disease or organ damage.	Treat within <24 hr. Perform surgery in cases of organ-threatening or life-threatening disease.
	Benign scrotal and penile surgery (orchidopexy, hydrocele, inguinal hernia, circumcision). Functional surgery (incontinence surgery, meatotomy, botulinum toxin injections). Genital reconstructive surgery (hypospadias, buried penis, other genital abnormalities). Benign (hemi) nephrectomy. Bladder augmentation, catheterizable stoma, appendicocecostomy due to the high and prolonged impact on patients and resources. Bladder exstrophy correction depending on age and local situation.	Surgery for VUR (open reimplant and bulk injection). Pyeloplasty if no loss of function. Urolithiasis if no infection or obstruction. Botulinum toxin injections for neurogenic bladder only in selected cases.	Pyeloplasty in UPJ obstruction with progressive loss of function or severe symptoms (consider drainage with JJ of nephrostomy). PUV. POM with progressive loss of function. Urolithiasis with recurrent infections.	Urosepsis with obstruction (urolithiasis, ureterocele with obstruction or POM). Trauma with hemodynamic instability or urinoma formation. PUV if urethral or suprapubic catheter cannot be placed. Oncology (Wilms, malignant testicular/paratesticular tumors, RMS of bladder and prostate, resection may be considered depending on local situation and condition of child). Acute ischemia (testicular torsion - in neonates not exploring is an option due to low chance of salvaging testis, very low risk of metachronous contralateral torsion, and increased vulnerability of these patients). Paraphimosis.
		**General considerations**		
While most children themselves may not be severely ill with COVID-19, this pandemic will impact pediatric urological care. Careful decisions must be made on what care requires postponement and what care is essential to be continued. Depending on the resources and capacity we recommend to only treat high-priority and emergency cases surgically during the COVID-19 pandemic. Consider treating intermediate-priority patients if capacity is available, but not during the COVID-19 surge. It is important to note that postponing surgery in patients with obstructive uropathy (UPJ, UVJ obstruction, PUV, neurogenic bladder) may lead to loss of renal function and the decision to postpone may be revised depending on the duration of the local situation as well as the severity of the obstruction in the individual case. Temporary drainage methods may be considered to bridge definitive surgery. Undoubtedly there will be cases of congenital abnormalities where the optimal surgical time point will be surpassed, such as hypospadias and cryptorchidism. These children may be at risk for suboptimal outcome or increased psychological burden due to delayed surgery and should be prioritized in the long waiting list.				

Abbreviations: **PUV** = posterior urethral valves; **POM** = primary obstructive megaureter; **UPJ** = ureteropelvic junction; **VCUG** = voiding cystourethrogram; **VUR** = vesicoureteral reflux; **UVJ** = ureterovesical junction; **and UTI** = urinary tract infection.

### EAU/ESPU Recommendations

Panels were asked to provide tables with recommendations based on the level of priority, including those that the panels felt were critical drivers of outcome and would especially be impacted by the current crisis, and always based on the highest level of evidence that was possible.

**LOW PRIORITY**: Clinical harm (very unlikely if postponed for 6 months).
**INTERMEDIATE PRIORITY**: Cancel procedure but reconsider if there is an increase in capacity (postponement for more than 3 months not recommended: clinical harm possible but unlikely if the procedure is postponed for more than 3 months).
**HIGH PRIORITY**: The last procedure to be cancelled; prevent delay of >6 weeks. Clinical harm (e.g., loss of organ function) very likely if the procedure is postponed for >6 weeks).
**EMERGENCY**: Cannot be postponed more than 24 hours. Life-threatening/organ function-threatening condition.

The BAPU also recommends that routine surgery be discontinued. Emergency surgery should be limited to category 4 or 5, unless local capacity is good enough to allow category 3 to be considered (15).

## ROUTINE PREOPERATIVE PCR IN CHILDREN

### Is PCR recommended in all children before any surgery?

The EAU/ESPU guidelines recommend performing PCR for the COVID-19 test prior to any surgical intervention whenever possible (16). Nasopharyngeal swab with RT-PCR performed within 48 h preoperatively for the detection of COVID-19 unfortunately shows a false negative rate of 30%-40% (17); however, it is always useful (18). If it cannot be performed or the test result is unknown, the patient is to be treated as positive and the number of personnel present in the operating room limited in order to reduce risks. Unfortunately, the literature regarding the effect of surgery on susceptibility to COVID-19 is very limited and relates only to adults. In one study, the mean age of 34 patients who underwent elective surgeries (levels 3 and 4) during the incubation period of COVID-19 was 55 years. All patients developed COVID-19 pneumonia shortly after surgery; 44.1% of the patients required admission to ICU during disease progression and 20.5% died after admission to ICU (19).

Regional or local anesthesia should be considered whenever possible to prevent the need for mechanical ventilation, although local anesthesia is very rare in children compared with adults (11).

### Risk of contagion in operating room situations

We know that certain procedures in the operating room generate aerosols (aerosol-generating procedures, AGP) and thereby increase the risk for surgical personnel if the patient is infected or in the incubation period (13). These include intubation, extubation, bronchoscopy, the introduction of chest tubes, electrocautery, and the use of ultrasonic devices. AGP should only be performed with full PPE, including an N95 mask or a powered air-purifying respirator (PAPR) designed for the operating room. It is advisable to use suction devices as much as possible.

### Laparoscopic/robotic/open surgical techniques

The European Association for Endoscopic Surgery reports that there is very little scientific evidence on the relative risks of minimally invasive surgery versus conventional open surgery in the context of COVID-19. However, it recommends that the risk of viral contamination of personnel during surgery, whether open, laparoscopic, or robotic, be considered and that protective measures be used strictly to ensure the safety of operating room personnel and to maintain a functioning workforce. For minimally invasive procedures, the use of devices to filter released CO2 for aerosol particles should be considered (20). While insufficient data are available to recommend for or against an open approach versus a laparoscopic/robotic approach, the surgical team must choose an approach that minimizes operating time and maximizes safety for both patients and staff (21).

In the Chinese experience, 3,387 healthcare workers were infected with COVID-19 with a mortality of 0.6%. In this setting, special caution is mandatory to reduce the infection among healthcare workers caring for COVID-19 patients. The EAU Robotic Urology Section (ERUS) has developed guidelines for robotic surgery during the COVID-19 emergency. In the case of nondeferrable surgery, the release of surgical smoke during laparoscopic procedures may carry small viral particles. As a consequence, any laparoscopic or robotic surgery should be performed only when necessary. It may be of particular importance to perform robotic surgery at the lowest permissible intraabdominal pressure (22).

As reported by Zheng et al. (23), ultrasonic scalpels or the electrical equipment commonly used in minimally invasive surgery can easily produce large amounts of surgical smoke, and in particular, the low-temperature aerosol from ultrasonic scalpels or scissors cannot effectively deactivate the cellular components of the virus in patients. These authors concluded that the particle concentration of the smoke in laparoscopic surgery is significantly higher than that in traditional open surgery (23, 24). Thus, it is recommended that lower electrocautery power settings be used as much as possible.

It is mandatory to confirm the complete and correct deflation of the pneumoperitoneum at the end of the procedure. In fact, due to the low gas mobility in the pneumoperitoneum, the aerosol formed during the operation tends to concentrate in the abdominal cavity. Sudden release of trocar valves, non-airtight exchange of instruments, or even small abdominal extraction incisions can potentially expose the health care team to the pneumoperitoneum aerosol. Therefore, CO2 should be aspirated as much as possible before removing trocars. In order to minimize the use of the operating room and optimize the use of surgical resources, procedures must be performed by experienced surgeons (20).

### Endoscopic procedures

Only one report in the literature has demonstrated the presence of SARS-COV-2 in urine specimens, in 6.9% of patients, and there is no available evidence on urine transmission (26). It is recommended that endoscopic procedures and urethral catheterization be performed with caution and that surgeons should be completely protected against infection if the patient has suspected or confirmed COVID-19.

## OUTPATIENTS AND TELEMEDICINE

To date, no specific treatment is available for COVID-19 infection and it is generally accepted that social distancing is the main and perhaps the only measure to prevent or contain the spread of infection so that the number of critical cases does not dramatically exceed the resources of a health system at risk of collapse. Reduction in outpatient clinic visits during various stages of severity of the COVID-19 pandemic is recommended. Pediatric urology telemedicine can lead to fewer patient contacts, lower infection rates among staff, and continuation of pediatric urological care by quarantined urologists. However, the proportion of patients eligible for telemedicine, their wish to use telemedicine, and their demographic risk profile for acquiring a severe pandemic infection are unknown. The ESPU has provided guidance on the reduction of outpatient clinic visits during the various stages of severity of the COVID-19 pandemic:

- **Stage 1:** Start to reduce outpatient cases such as benign scrotal and penile pathology as well as incontinence.
- **Stage 2**: See only cases that are at least semi urgent, such as those requiring initial postoperative ultrasound after upper tract reconstruction. Consider postponing prolonged (postoperative) follow-up in stable patients.
- **Stage 3**: Continue care for urgent cases in which delay may cause irreversible progression of disease or organ damage. This includes ultrasound and voiding cystography in suspected severely obstructive uropathy in which surgery is still considered.
- **Stage 4**: Continue all care for cases in which a delay of care is potentially organ-threatening or life-threatening.

In the case of postoperative follow-up of patients with genitourinary pathologies, it is advisable to carry out the follow-up by sending photographic documentation in compliance with the General Data Protection Regulation (GDPR). If the visit has to be in person, the patient should be accompanied by a single caregiver (14). A distance of 2 m should be maintained between patients. Every child with suspected respiratory infection should wear a mask. Children under one year of age must be kept in their strollers and in baby seats or restraint systems and away from other patients. In pediatric waiting rooms, there will be no materials such as toys, books, or other objects that children can share and that cannot guarantee that recommended material hygiene and cleanliness standards are met, in addition to evidence of transmission before the manifestation of symptoms. If there are COVID-19 symptoms, the child or caregiver has tested positive for COVID-19, or they are in quarantine, they should be seen in a COVID-dedicated area of the hospital without interaction with other patients (8).

## TRAINING PROGRAMS

All interhospital staff movements with residents training in other hospitals and all undergraduate clinical rounds have been canceled. All training programs for residents as well as fellowship programs in pediatric urology in Spain have been affected. Many residents have had to become so-called front-line doctors caring for patients affected by COVID-19. It is recommended that all procedures are performed by experienced urologists confident in the procedure. Procedures should be performed with the minimum number of staff members, who should also be fully trained and experienced. Furthermore, no external observers (i.e., fellows or students) should be present during procedures until the pandemic has been controlled, which we hope will be in the approaching period (22). Currently, training meetings held between companies or for the same department are scheduled via telematics.

## INCREASING SURGICAL ACTIVITY AFTER THE PANDEMIC IS OVER

There is no existing knowledge on the adverse impacts of loss of surgical capacity on patients' surgical condition and associated health or on prognosis. A new model will have to be established after the pandemic based on the length of the surgical waiting list.

## WHAT ABOUT LATIN AMERICA? WHAT HAVE THEY LEARNED FROM EUROPE'S EXPERIENCE?

Countries in Latin America are following the programs applied in Europe because the European countries have more experience with COVID-19. In preparation for potential surges in cases of COVID-19, most governments have chosen to create new healthcare facilities and have emphasized the need for careful planning around elective procedures, taking into account multiple considerations such as adequacy of supplies of PPE and other essential equipment, testing capacity, sanitation protocols, and workforce availability. Hospitals need to maintain adequate staffing levels to cover a potential surge in COVID-19 cases and should have enough beds, PPE, ventilators, and trained staff to allow these surgeries to take place without resorting to a crisis standard of care.

Elective surgeries were initially suspended to preserve hospital bed capacity and PPE. When the data indicate a better position regarding hospital capacity, and provided individual institutions can accommodate their internal demand for PPE, it may be time to start performing some of these procedures again.

As in many countries, training programs have continued through societies, webinars, and virtual masterclasses.

Across Latin America, and indeed in all developing countries facing the COVID-19 pandemic, there are many unanswered key questions relating to impacts on the economy, levels of poverty, social and psychological problems, crime post quarantine, etc. No nation is prepared to face this crisis, but in developing countries the problem is even worse because they are all constantly in a state of crisis. In this context the post-COVID-19 era represents a huge challenge.

## CONCLUSIONS

The COVID-19 virus has been impacting dramatically on the normal life of the departments. Because of the necessity to adopt strategies to contain the diffusion, all surgical departments have to be restricted. Perform surgery only in cases of organ-threatening or life-threatening disease. Suggested reduction in outpatient clinic visits during various stages of severity of the COVID-19 pandemic.

## SUMMARY OF RECOMMENDATIONS

- Consider treating only high-priority and emergency cases surgically during the COVID pandemic.
- Consider treating intermediate-priority patients if capacity is available but not during the COVID surge.
- Non-surgical management should be considered, to begin with, including medical treatment (e.g. antibiotics for vesico-ureteral reflux associated urinary tract infections), endovascular embolization (e.g. for bleeding renal traumas), or urinary tract diversion.
- Perform PCR for the COVID-19 test prior to any surgical intervention whenever possible.
- Follow the local recommendations for personal protective equipment (PPE).
- Avoid or reduce the use of monopolar electrosurgery, ultrasonic dissectors, and advanced bipolar devices, as these can lead to particle aerosolization.
- All minimally invasive procedures should preferably be performed by experienced surgeons.
- It is recommended that electrocautery power setting be lowered as much as possible in order to reduce the surgical smoke production, especially in laparoscopic surgery. During access, electrocautery should be provided with automatic suction system.
- Reduction in outpatient clinic visits during various stages of severity of the COVID-19 pandemic is recommended
- Multidisciplinary team meetings are recommended to offer the optimum therapeutics.
- Regional or local anesthesia should be considered whenever possible to prevent the need for mechanical ventilation.

## ABBREVIATIONS

PCR: Polymerase Chain Reaction
BAPU: British Association of Pediatrics Urologist
PPE: Personal Protective Equipment
